# Genomic analysis of three African strains of *Bacillus anthracis* demonstrates that they are part of the clonal expansion of an exclusively pathogenic bacterium

**DOI:** 10.1002/nmi2.62

**Published:** 2014-09-28

**Authors:** L Rouli, M MBengue, C Robert, M Ndiaye, B La Scola, D Raoult

**Affiliations:** 1 Aix Marseille Université, URMITE, UM63, CNRS 7278, IRD 198, Inserm 1095Marseille, France; 2 Laboratoire National d'Elevage et des Recherches Vétérinaires (LNERV), Institut Sénégalais de Recherches Agricoles (ISRA)Hann, Dakar, Senegal; 3 Laboratoire de Biologie Cellulaire, Faculté des Sciences et Techniques de l'Université Cheikh Anta DIOP de Dakar (UCAD)Dakar, Senegal

**Keywords:** African strains, *Bacillus anthracis*, pan-genome, pathogen, specialization

## Abstract

*Bacillus anthracis* is the causative agent of anthrax and is classified as a ‘Category A’ biological weapon. Six complete genomes of *B. anthracis* (A0248, Ames, Ames Ancestor, CDC684, H0491, and Sterne) are currently available. In this report, we add three African strain genomes: Sen2Col2, Sen3 and Gmb1. To study the pan-genome of *B. anthracis*, we used bioinformatics tools, such as Cluster of Orthologous Groups, and performed phylogenetic analysis. We found that the three African strains contained the pX01 and pX02 plasmids, the nonsense mutation in the *plcR* gene and the four known prophages. These strains are most similar to the CDC684 strain and belong to the A cluster. We estimated that the *B. anthracis* pan-genome has 2893 core genes (99% of the genome size) and 85 accessory genes. We validated the hypothesis that *B. anthracis* has a closed pan-genome and found that the three African strains carry the two plasmids associated with bacterial virulence. The pan-genome nature of *B. anthracis* confirms its lack of exchange (similar to *Clostridium tetani*) and supports its exclusively pathogenic role, despite its survival in the environment. Moreover, thanks to the study of the core content single nucleotide polymorphisms, we can see that our three African strains diverged very recently from the other *B. anthracis* strains.

## Introduction

Blackleg, or anthrax, was the first disease to be linked to a specific microbe by Davaine in 1863 and the first animal infection for which a vaccine was proposed (by Pasteur, 1881) 1. In 1876, Koch discovered the first bacterium, *Bacillus anthracis*, which had the capacity to transform into spores 1*. Bacillus anthracis* belongs to the phylum Firmicutes and is part of the *Bacillus cereus* group 2. It is a Gram-positive, spore-forming soil bacterium 2 that is a facultative anaerobe 3, able to survive in extreme and unfriendly environmental conditions, such as high levels of radiation or extreme temperature 2, and remain viable in the soil over a long period of time 4. *Bacillus anthracis* is the causative agent of the zoonotic disease anthrax 2, to which cattle and horses are particularly sensitive 1. Human infections can occur through the ingestion or inhalation of spores or through contact with the skin 2. Four clinical syndromes for anthrax disease exist 4: cutaneous anthrax (95% of reported cases), gastrointestinal anthrax due to contaminated food, inhalational anthrax and injectional anthrax. *Bacillus anthracis* is classified as a ‘Category A’ biological weapon 2. Due to the stability of its spores, its high pathogenicity and lethality, and its capacity to be inhaled 5, the weaponization of *B. anthracis* by US 1 and Soviet armed forces has led to two series of cases, as reported by a US Army investigator and identified through genomic analysis 6. Additionally, an anthrax epidemic caused by atmospheric contamination from a military laboratory occurred in 2001 1,7, leading to an increase in research about *B. anthracis* and anthrax 5 and prompting the emergence of a new detection system 2.

Compared with *B. cereus*, *B. anthracis* has a nonsense mutation in the *plcR* gene at position 640 7 and four prophage regions. *Bacillus anthracis* strains can be classified into three different lineages (A, B or C) 8 based on multilocus variable number tandem repeat (VNTR) analysis (MLVA) 9–11. Lineage A, which is found worldwide, is divided into four sub-lineages: A1, A2, A3 and A4. Lineage B is divided into two sub-lineages, B1 and B2, with B1 found in southern Africa and B2 in southern and Eastern Europe. Lineage C is rare.

The first pan-genomic study was published in 2005 on *Streptococcus agalactiae* 9, and the number of such studies has increased rapidly since then. A pan-genome is defined as the pool of all genes present in all the studied genomes of a species, which allows for a comparison between different species or strains. The pan-genome can be divided into different parts: the core genome (genes present in all the genomes), accessory genes (genes present in several genomes but not all) and unique genes (genes present in only one of the studied isolates). Additionally, the pan-genome can be closed or open, depending on the capacity of the species to acquire new genes 9 and the age of the initial clone. The capacity of the species to gain genes can be evaluated by studying its mobilome. Its age can be determined using the single nucleotide polymorphism (SNP) observation process in the core genome 10–12.

African strains of *B. anthracis* from Gambia and Senegal have not yet been studied 13. In this study, we compiled the *B. anthracis* pan-genome based on three African strains (two from Senegal and one from Gambia) and six reference genomes (Ames 14, Ames Ancestor 12, A0248, CDC684 15, H9401 13 and Sterne). The Ames strain is the non-virulent version of the Ames Ancestor strain and was the strain found in letters in the USA in 2001. Virulence is linked to the presence of two plasmids, pX01 and pX02, which carry genes encoding for toxins, as well as the capsule 4. Our work demonstrates that the three African strains are very closely related to the other strains, especially CDC684, contain the pX01 and pX02 plasmids, belong to the worldwide lineage A and have a closed pan-genome.

## Materials and Methods

### Bacteriological studies

Organ harvesting was conducted at different sites according to biosecurity procedures. Cultures were grown in a liquid medium consisting of a nutrient broth (Liofilchem s.r.l., #610037, Roseto degli Abruzzi, Italy). The bacteriological study procedure included the following steps. After seeding on trypticase soy agar (Bio-Rad, #64946, Marnes-la-Coquette, France), the bacterial mixture was incubated at 37°C for 24 h. The bacteria were then streaked on Columbia sheep blood agar (blood culture) and incubated as previously described. Isolates were assessed for Gram positivity using Gram staining and were seeded for biochemical characteristic studies.

Eight isolates suspected as possibly being *B. anthracis* were sent to the URMITE laboratory. Identification was performed using a system based on the molecular detection of the *pag* gene under previously described conditions 14. The production of acetyl methyl carbinol, as well as the fermentation of certain sugars, was assessed on isolates identified as *B. anthracis,* using previously reported methods 15. Cultural, morphological and biochemical characteristics were studied in detail using conventional methods. The experimental pathogenicity of the *B. anthracis* strains was assessed using strain BALB/c white mice (age 6 months). These mice were offered by the Pasteur Laboratory institute of Dakar. Intraperitoneal inoculations of 500 μL of the isolated *B. anthracis* cultures was administered (culture at 104 CFU/mL), and the animals were monitored for 24 h. Autopsies were performed post-mortem, and Giemsa-stained smears of the spleen were observed using a microscope at 100 × magnification after immersion.

In the pathogenicity experiments that were performed on white mice, two experimental mice received 500 μL of the bacterial culture intraperitoneally and two control mice received only 500 μL of physiological buffer. These mice were monitored in the laboratory.

When the mice died, an autopsy was performed and *B. anthracis* was identified by examining Giemsa-stained smears of the spleen 15.

### Sequencing

Sequencing of the Sen2col2, Sen3 and Gmb1 strains of *B. anthracis* were performed using the SOLiD 4_Life Technology's New Generation Sequencing technology. The paired end library was constructed from 1 μg of purified genomic DNA from each strain. The sequencing was carried out to 50 × 35 base pairs (bp) using SOLiD™ V4 chemistry on one full slide associated with 96 other projects on an Applied Biosystems SOLiD 4 machine (Applied Biosystems, Foster City, CA, USA). All 96 genomic DNA samples were barcoded with the module 1–96 barcodes provided by Life Technologies (Paisley, UK). The libraries were pooled in equimolar ratios, and emPCR (PCR by emulsion) was performed according to the manufacturer's specifications, using templated bead preparation kits on the EZ bead automated Emulsifier, Amplifier and Enricher E80 system for full-scale coverage. A total of 708 million P2-positive beads were loaded onto the flow cell for the run, and the output read length was 85 bp, as expected (50 × 35 bp). The three *B. anthracis* genomes (Sen2col, Sen3 and Gmb1) were sequenced through 3.2^E^ + 6, 3.1^E^ + 6 and 3.9^E^ + 6 barcode reads, which led to 273, 262 and 382 Mb of data, respectively. The global sequencing of these three genomes resulted in 917 Mb of data.

For each of the three strains, we performed a mapping against the Ames reference strain though CLC workbench software. For mapping we used relatively stringent parameters (length fraction of 0.7 and similarity fraction of 0.8).

### Basic genomic data

The complete genomic sequences of the six references strains are available on NCBI: Ames 16 (NC_003997.3), Ames Ancestor 12 (NC_007530.2), A0248 (NC_012659.1), CDC684 17 (NC_012581.1), H9401 13 (NC_017729.1) and Sterne (NC_005945.1). The sequences of the plasmids pX01 and pX02 from A0248, Ames Ancestor, CDC684 and H9401 are also available on NCBI: NC_012656.1, NC_012655.1, NC_007322.2, NC_007323.3, NC_012579.1, NC_012577.1, NC_017726.1, NC_017727.1, respectively. H9401 came from Korea, Sterne came from the UK, and the four other strains came from the USA. Our strains of interest came from Senegal (Sen2Col2 [PRJEB1516] and Sen3 [PRJEB1517]) and Gambia (Gmb1 [PRJEB1518]) and were isolated in 2010. Sen2Col2 was isolated from the lungs of a 6-year-old ostrich. Sen3 was isolated from the lungs, liver, spleen and blood of a Touabire sheep. Finally, Gmb1 was isolated from the blood of a trypanotolerant zebu cow. The sequences of these three African strains and their plasmids were obtained from the SOLiD data.

### Genomic analysis

#### Determination of gene functions and the B. anthracis mobilome

To study genomic content and perform functional analysis, we first used CAMERA 18, a bioinformatics portal that can perform several types of analysis. In this portal, we performed cluster of orthologous groups (COG) analysis 19 to assign functional annotations to proteins, which were classified into categories (the list is available at http://www.ncbi.nlm.nih.gov/COG/old/palox.cgi?fun=all). Next, we investigated metabolic pathways using the Kyoto Encyclopedia of Genes and Genomes 20 data and the data generated KEGG by the KAAS 21 (KEGG Automatic Annotation Server) online tool. In KEGG, the proteins were classified into classes and subclasses. We also used RAST 22 to annotate the new strains and their plasmids and to find the mobilome. Regarding the mobilome, we also used CRISPRfinder 23 and PHAST 24. We used MeV 25,26 (Multi Experiment Viewer) to best visualize the distribution of the accessory genes and to perform hierarchical clustering on the COG and KEGG data.

#### Phylogeny, MLVA and orthology

We performed a global genome alignment using MAUVE 27 and a multiple alignment with MEGA5 28 (using the ClustalW algorithm), followed by a tree reconstruction (distance or neighbour joining method). Using MAUVE and its backbone output file, we calculated the ratio of the size of the core genome to that of the pan-genome to evaluate the closed or open nature of the pan-genome.

We were also interested in determining the lineages of the three African strains. For lineage analysis, we used the MLVA8 system 10, which is composed of eight VNTR loci: *vrrA*, *vrrB1*, *vrrB2*, *vrrC1*, *vrrC2, CG3, pXO1-AAT* and *pXO2-AT* 29. The NCBI and the MLVA databases were also helpful for lineage analysis.

Next, we used OrthoMCL 30 with default parameters to obtain a list of orthologs and determine the pan-genome composition (core, accessory and unique genes) of *B. anthracis*. We also investigated the SNPs present in the core genome by phylogeny, performing a maximum likelihood tree (phyml) with 100 bootstrap iterations. For the core genome tree and the transition/transversion bias, we used MEGA5. Finally, based on the core SNPs tree, we created a time tree based on the Reltime method, developed by Tamura *et al*. in 2012 31. This tree allowed the estimation of the divergence time in millions years and was built using the MEGA 6 software.

## Results

### General features and phylogeny

**Figure 1:**
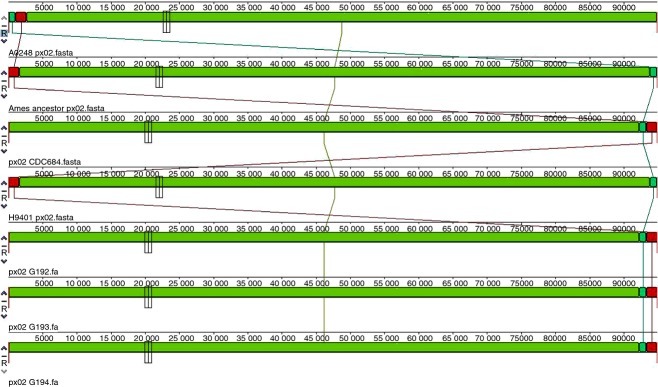
G192: Sen2Col2, G193: Sen3 and G194: Gmb1. MAUVE global alignment on pX02 plasmids.

**Figure 2:**
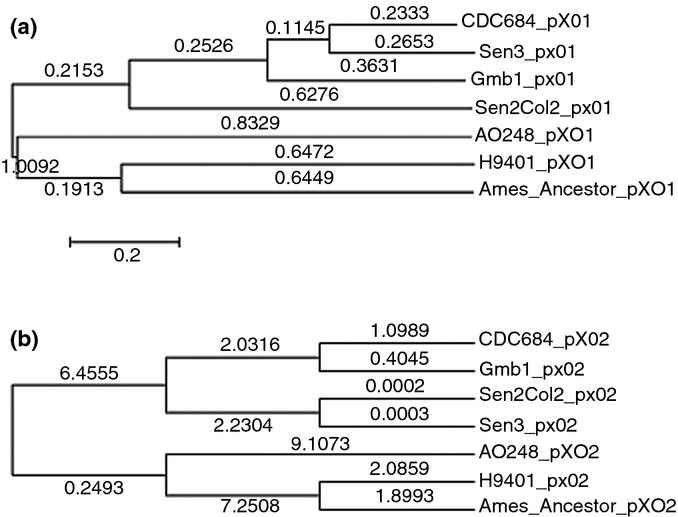
Numbers on the branches correspond to branch length. (a) Tree based on the pX01 plasmid. (b) Tree based on the pX02 plasmid.

For each of the three strains, we obtained more than 6 million reads from the SOLiD sequencing. Average coverage was approximately 30, and maximum coverage could reach 3000. The genomes of the three African *B. anthracis* strains, Sen2Col2, Sen3 and Gmb1, were the same size as the other known strains (5.23 Mb), and they had the same average number of proteins (approximately 5300) and the same G+C% value. The three genomes were deposited at EMBL with the accession numbers CAVC010000000, CAVD010000000 and CAVE010000000, respectively. The MAUVE alignment revealed no rearrangements and a high conservation among all genomes. Examination of the *plcR* gene revealed a nonsense mutation typical to *B. anthracis* 7 at position 640 and the four known prophage regions in the three new strains. The low values of the branches on the phylogenetic tree (not shown) indicated high similarities between the strains. We also examined the *rpoB* gene but could not perform a phylogenetic analysis because the sequences of all nine strains were identical. Regarding the plasmids, we found that pX01 and pX02 in the three Africans strains were the same size (0.18 Mb and 0.094 Mb, respectively) and contained the same average number of genes (195 and 102, respectively). There were no gaps in the new plasmid sequences. The MAUVE alignments (Fig.1) illustrate the similarities between the plasmids. The pX01 plasmids from CDC684 and the three African strains exhibit an inversion, and the pX02 plasmids of the three African strains are identical to that of CDC684. Additionally, based on the analysis of the phylogenetic trees (Fig.2a,b), the two plasmids of the African strains are closely related to those from strain CDC684.

**Figure 3:**
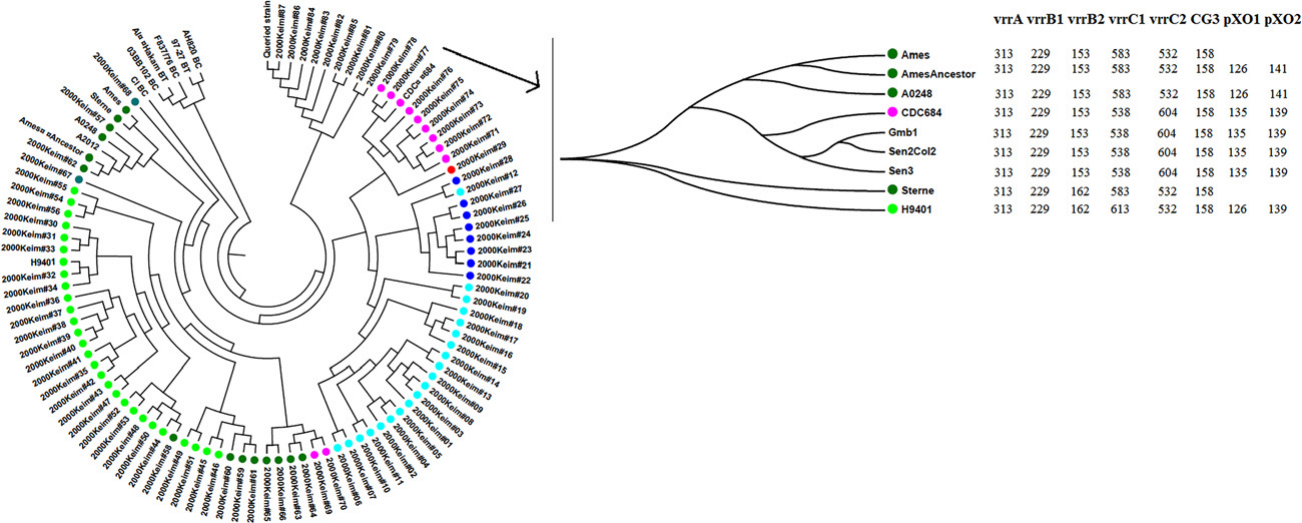
Multi locus VNTR analysis tree. Colours correspond to lineages: light blue, A1a; dark blue, A1b; red, A2; light green, A3a; dark green, A3b; blue green, other A3; pink, A4.

We defined the profile of the eight VNTR loci for the three new strains, and the tree shows that these African strains again clustered with CDC684 (Fig.3). Using the values of the eight loci and comparing them to the work of Keim et al. 10, we found that the three African strains belong to lineage A. More specifically, we found that these strains belong to lineage A4, the same lineage as CDC684 and one other African strain 10.

### Genomic comparison

#### KEGG and COG

**Figure 4:**
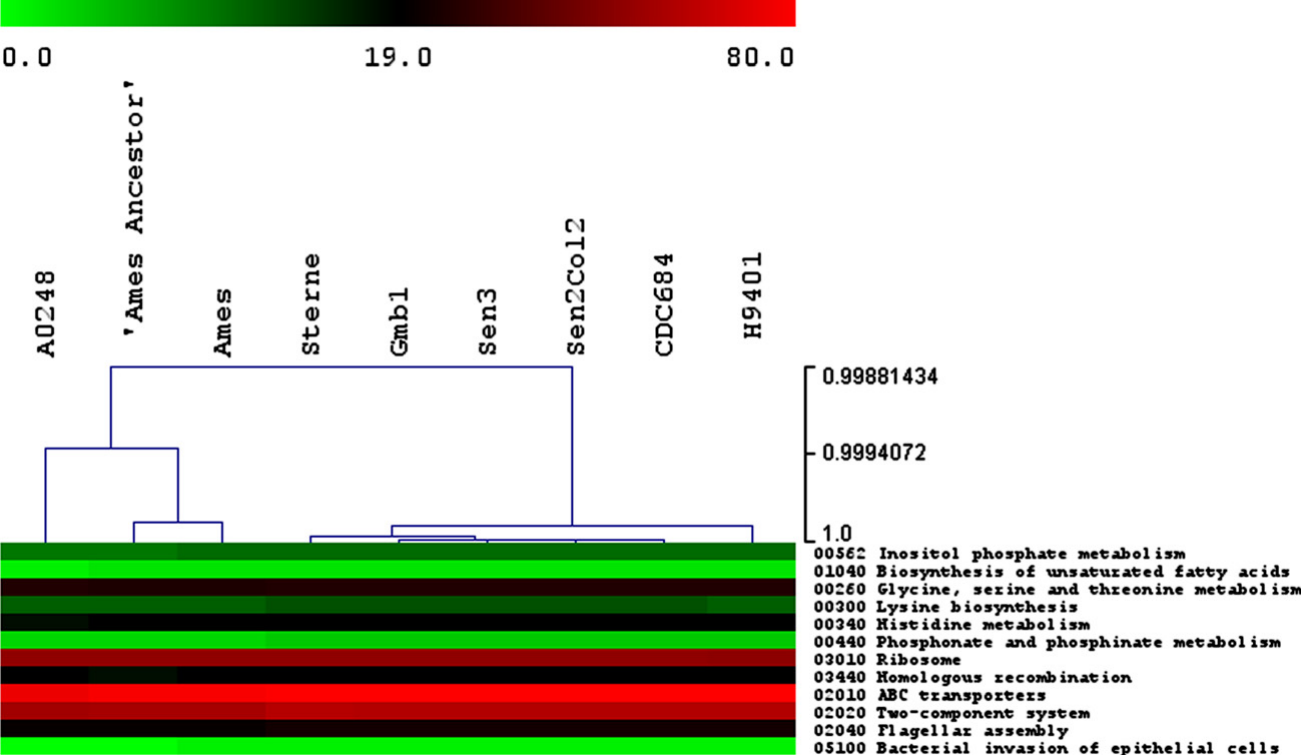
Analysis of Kyoto Encyclopaedia of Genes and Genomes (KEGG) results. Hierarchical clustering of the strains based only on the difference in the KEGG distribution. Colours depend on the number of proteins implied in each category for each strain. The scale is presented in the figure.

The three African strains are identical to each other and to CDC684 at a functional level. Fig.4 represents the clustering of the strains based only on the difference in the KEGG distribution. Divergences are as follows: one difference with Sterne (ko02020), two with H9401 (ko03010 and ko00300), and multiple differences with Ames and Ames Ancestor (ko00260, ko00300, ko00440, ko02010, ko02020, ko02040), and A0248 (ko00562, ko01040, ko00300, ko00340, ko00440, ko02010, ko02020, ko02040, ko05100). When we examined these details, we noticed two groups: one group contains A0248, Ames and Ames Ancestor, and the other group contains the other strains. There were no differences between the pX01 and pX02 plasmids of the African strains and those of the other strains (data not shown). We looked in detail at categories related to the genetic systems for DNA surveillance because they might be a part of the slow evolution of these bacteria. DNA replication and nucleotide excision repair were complete and active. With regard to homologous recombination, the ruvC gene is absent in all strains. For mismatch repair, mutH, the unique enzyme for the incision step, is absent. Hence, this pathway is incomplete.

**Figure 5:**
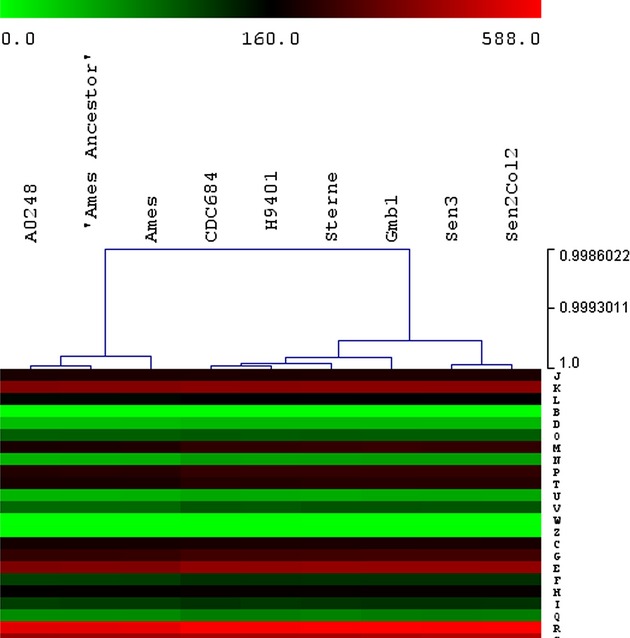
Clustering of the strains based on the distribution of all the Cluster of Orthologous Groups categories. J, translation, ribosomal structure and biogenesis; K, transcription; L, replication, recombination and repair; B, chromatin structure and dynamics; D, cell cycle control, cell division, chromosome partitioning; O, post-translational modification, protein turnover, chaperones; M, cell wall/membrane/envelope biogenesis; N, cell motility and secretion; P, inorganic ion transport and metabolism; T, signal transduction mechanisms; U, intracellular trafficking, secretion and vesicular transport; V, defence mechanisms; W, extracellular structures; Z, cytoskeleton; C, energy production and conversion; G, carbohydrate transport and metabolism; E, amino acid transport and metabolism; F, nucleotide transport and metabolism; H, coenzyme transport and metabolism; I, lipid transport and metabolism; Q, secondary metabolite biosynthesis, transport and catabolism; R, general function prediction only; S, function unknown. Colours depend on the number of proteins implied in each category for each strain. The scale is presented in the figure.

The COG data showed similar results. First, as shown in Fig.5, a cluster with Ames, Ames Ancestor and A0248 (group 1) and another cluster with the other strains (group 2) was found. Moreover, we can see uniformity between the strains inside each of the COG categories, except in the E (amino acid transport and metabolism), G (carbohydrate transport and metabolism), P (inorganic ion transport and metabolism) and M (cell wall/membrane/envelope biogenesis) categories, where there is, for group 1, approximately 5% less protein function as group 2. These small differences can be observed as slight modifications of the colours in Fig.5.

#### Pan-genome, SNPs and mobilome

The mobilome was examined, and the same trend was found in all of the strains: two transposases, one or two confirmed CRISPRs and numerous proteins/associated proteins from phages or prophages. We also examined the mobilome in the plasmids. All of the pX01 plasmids contained five transposases, one phage and no CRISPRs. All of the pX02 plasmids contained two transposases and no CRISPRs.

**Figure 6:**
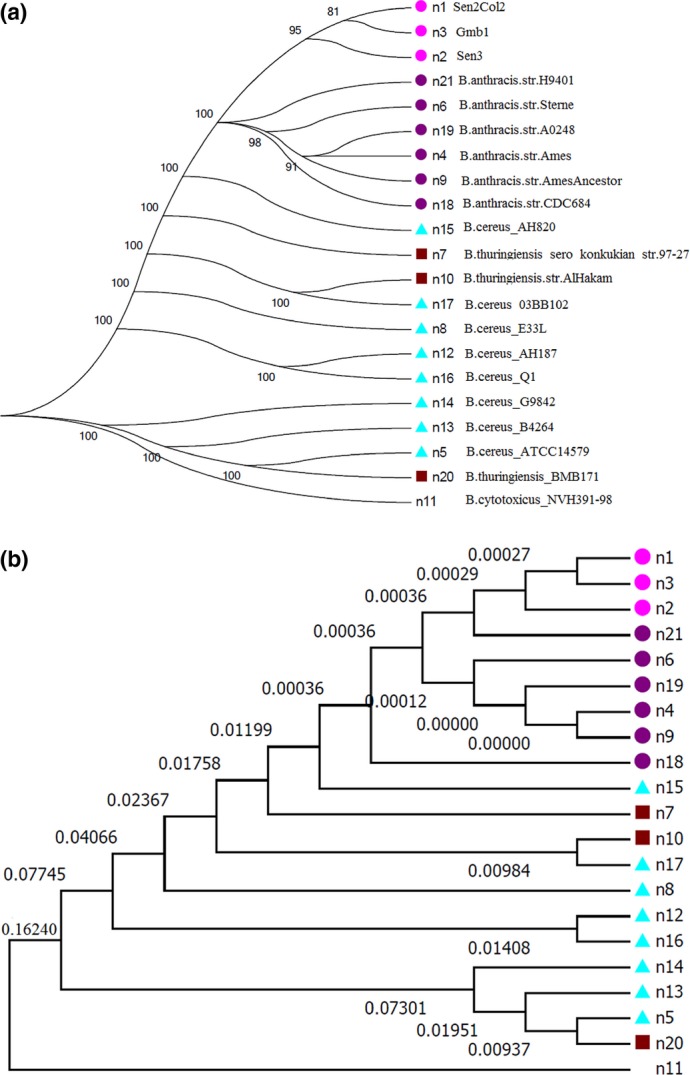
Single nucleotide polymorphisms of the core content based tree. The percentage on the branches corresponds to bootstraps. This is a PhyML tree with 100 bootstrap iterations. Colours correspond to the species. Purple is *Bacillus anthracis*, pink is our three African *B. anthracis* strains, blue is *Bacillus cereus* and brown is *Bacillus thuringiensis*. (a) Numbers on branches correspond to bootstrap values. (b) Time tree based on the previous tree. Numbers on the branches are the divergence time, given in million years.

The pan-genome is composed of 2893 core genes, seven unique genes and 85 accessory genes. First, we considered the unique genes. We found five in Sterne (two not found on NCBI, one conserved hypothetical protein, an EmrB/QacA family drug resistance transporter and a zinc-binding dehydrogenase), one in CDC684 (not found on NCBI) and one in H9401 (a yfeT DNA-binding transcriptional regulator), but all were false positives, as they were eventually identified in other *B. anthracis* strains available on the NCBI database. Next, we carefully examined the 85 accessory genes. The three African strains and CDC684 contained almost all of the accessory genes. Half of the accessory genes were annotated as hypothetical proteins. The hierarchical clustering again resulted in the same two groups found using COG and KEGG (one with Ames, Ames Ancestor and A0248 and the second with the other strains). Moreover, we calculated the core/pan-genome ratio and found that the core genome represented 99% of the pan-genome (Table1), again indicating the high rate of conservation among the nine strains. Finally, we studied the SNPs at the core genomic level. We found approximately 3800 SNPs between the nine strains, with 2911 in the core. Among the three African strains, there are 1500 SNPs in the core and 1120 among the six reference strains. We can see that the three African strains cluster together. Moreover, we have a transition/transversion bias of 0.32 between the nine strains. The very small rate of SNPs, low transition/transversion bias and very high ratio of the core genome function to the pan-genome indicates that *B. anthracis* evolves very slowly. To go further, we generated a maximum-likelihood tree based on SNPs of the core of the nine *B. anthracis* strains, together with eight *B. cereus*, three *Bacillus thuringiensis* and, as an outgroup, one *Bacillus cytotoxicus* (Fig.6a) strain. This tree clearly showed that our three African strains evolved differently from the other six strains of *B. anthracis* and that is an ancestral difference. Moreover, we obtained good bootstraps, so we could be confident about this cluster. Finally, we generated a time tree (Fig.6b) to estimate the divergence time between each cluster. On Fig.6b, the number on branches are indicated in millions of years. For instance, between the African cluster and *B. anthracis* H9401, the estimated divergence time is approximately 360 years. Moreover, the estimated divergence time between the *B. anthracis* cluster and *B. cereus* AH820 (n15 on Fig.6b) is approximately 12 000 years. For comparison, in a work in 1999, Achtman *et al*. first estimated the divergence time between *Yersinia pestis* and *Yersinia pseudotuberculosis* to be between 1500 and 20 000 years. Then, in 2004 32, they looked at different pathovars of *Y. pestis*. They found the estimated divergence time between an Orientalis strain (CO92) and a Medievalis strain (KIM) to be approximately 6500 years.

**Table 1:** Pan-genomes of human clonal pathogens

Species	Genome used	Lifestyle	Intracellular	Niche	%
*Bacillus anthracis*	9	Allopatric	No	Soil	99
*Rickettsia rickettsii*	8	Allopatric	Yes	Ticks	99
*Chlamydia trachomatis*	20	Allopatric	Yes	Human	99
*Rickettsia prowazekii*	8	Allopatric	Yes	Human	100

The % column corresponds to the core/pan-genome ratio.

## Discussion

**Figure 7:**
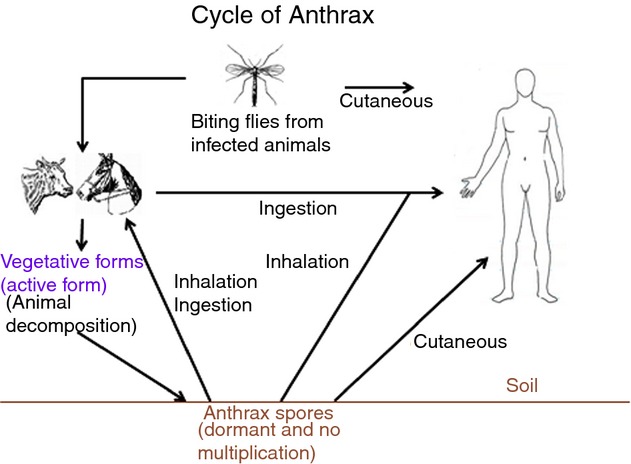
Summary of the *Bacillus anthracis* lifestyle.

In comparing the three African strains to the other *B. anthracis* strains, we noticed that all of the African strains carried the two virulence plasmids (pX01 and pX02), the *plcR* nonsense mutation and four prophages. We also determined that these strains were more closely related to CDC684 at the functional, sequence, plasmid and lineage levels. In this work, we validate the finding that, as previously shown 9, the pan-genome of *B. anthracis* is very narrow and that *B. anthracis* is clonal. To have confidence in our study, we used many different tools to compare and validate our results. All of the tools yielded the same results: the addition of the three African strains did not change the nature of the *B. anthracis* pan-genome (2893 core genes and 85 accessory genes), which had a core/pan-genome ratio of 99%. This core/pan-genome ratio is very close to those from the other human clonal pathogens (Table1), such as *Rickettsia rickettsii*. However, there is discordance between the presence of a mobilome (some phages) and the fact that *B. anthracis* has a closed pan-genome. Nevertheless, *B. anthracis* is derived from the *B. cereus* group, a sympatric species that is not intracellular. Therefore, *B. anthracis* may have become entirely allopatric. Moreover, based on the core genome, we found very few SNPs (896), a very small transition/transversion bias (0.32) and no CRISPRs. Hence, we can hypothesize that *B. anthracis* is an ancient clone that evolved slowly. Indeed, the work of Mancini and Ippolito 33 describes the history of anthrax disease and suggested that the first case may date back to antiquity. However, calculating the age of this type of bacterium is difficult due to its life cycle (a short vegetative phase of 20–40 generations and a long spore phase) 9. *Bacillus anthracis* does not replicate in the spore phase, and when it is in tissues, it may replicate as a pathogen to avoid living in sympatry. This behaviour is similar to *Clostridium tetani* 34, a sporulating, anaerobic bacterium that resides in the soil and is pathogenic for humans and animals. We recently obtained a new genome of *C. tetani* and found that it is also very close to the reference genome 35. Therefore, the *B. anthracis* pathogen evolves very slowly compared with other species with similar generation times 18,19. We believe that the lack of gene transfer and defence mechanisms (CRISPRs) observed in intracellular bacteria 36 suggests that *B. anthracis* multiplies only as a pathogen and that its life in soil is exclusively dormant (Fig.7).

There are different types of pathogens. Opportunist pathogens, such as *Pseudomonas aeruginosa*, live in the environment but are not specialized. Real pathogens, such as *B. anthracis*, *Y. pestis* 37, *C. tetani* and *Tropheryma whipplei*, are specialized and have a small and closed pan-genome. Each of these pathogens has a core/pan-genome ratio of more than 90%. Real pathogens can survive in the soil or water but cannot multiply outside their niche. A pan-genome study, as opposed to a virulence study, can help to determine if a species is a real, specialized pathogen.

## Conclusion

Due to the lengthy spore phase of its life cycle, *B. anthracis* evolved very slowly and has a very narrow pan-genome, despite its apparent soil ecological niche. We found that the three African strains examined belong to lineage A (worldwide lineage), specifically lineage A4, similar to CDC684 and another previously characterized African strain. Pan-genome analysis allowed us to assess the lifestyle of this pathogen and confirmed its allopatric, highly specialized lifestyle. Our studied African strains diverged very recently from the other *B. anthracis* strains.
